# Implementation of Nursing Process and Its Association with Working Environment and Knowledge in Ethiopia: A Systematic Review and Meta-Analysis

**DOI:** 10.1155/2020/6504893

**Published:** 2020-07-18

**Authors:** Wondimeneh Shibabaw Shiferaw, Tadesse Yirga Akalu, Abate Dargie Wubetu, Yared Asmare Aynalem

**Affiliations:** Department of Nursing, College of Health Science, Debre Berhan University, Debre Berhan, Ethiopia

## Abstract

**Background:**

The nursing process is a scientific problem-solving approach, which directs nursing care and potentially improves quality of health care service. The national pooled implementation of the nursing process in Ethiopia remains unknown. Hence, this review and meta-analysis aimed to estimate the overall implementation of the nursing process and its association with the working environment and knowledge in Ethiopia.

**Methods:**

PubMed, Scopus, Cochrane Library, Google Scholar, PsycINFO, and CINAHL were searched and complemented by manual searches. The DerSimonian and Laird random effects model was applied to estimate the pooled effect size, odds ratios, and 95% confidence interval across studies. The *I*^2^ statistic was used to check heterogeneity between the studies. Sensitivity analysis was deployed to see the effect of a single study on the overall estimation. Publication bias was examined using funnel plot and Egger's regression test statistic. Analysis was performed using STATA™ Version 14 software.

**Results:**

Seven studies comprised of 1,268 study participants were included in this meta-analysis. The estimated pooled implementation of the nursing process in Ethiopia was 42.44% (95% CI: 36.91, 47.97%). Based on subgroup analysis, methods of outcome measurement showed that the highest overall implementation of the nursing process was observed from studies conducted using self-report technique 42.95% (95% CI: 35.76, 50.15). Nurses working in stressful environment were 81% less likely to implement the nursing process (OR 0.19, 95% CI: 0.04, 0.76), and nurses having good knowledge were 8 times more likely to implement nursing process (OR 8.38, 95% CI: 2.82, 24.86).

**Conclusion:**

The overall implementation of the nursing process in Ethiopia was relatively low. Good knowledge of nurse had paramount benefits to improve implementation of the nursing process. Therefore, nurse can be educated on the imperative of knowledge in order to enhance the nursing process implementation and to improve the overall quality of healthcare services. Furthermore, policymakers and other concerned bodies should give special attention to improving the implementation of the nursing process.

## 1. Background

Nursing is a dynamic profession with a unique perspective on people, environment, and health [1]. The nursing process is a systematic problem-solving approach used to identify, prevent, and treat actual or potential health problems and promote wellness. It consists of five steps: assessment, diagnosis, planning, implementation, and evaluation [2]. The use of the nursing process helps in making and planning a clear and effective nursing care that potentiates improvement of the quality of patient care [3]. Implementation of the nursing process in clinical settings facilitates high quality nursing care, improves client health outcomes, and promotes nursing as a professional scientific discipline [4]. In addition, studies claim that, by implementing the nursing process, the nursing profession will be strengthened, internationalized, and dignified as efforts to achieve patient care criteria [5]. The approach of client care has moved from the medical to a holistic care model [1]. The nursing process, in its emphasis on patient-centred and goal-oriented care, has the potential to improve the quality of nursing care and to meet individualized health care needs [6–10].

Emphasis on holistic patient care within the nursing process is key to delivering quality nursing practice and central to nursing education [11]. Standard implementation of the nursing process could improve quality of care and encourages the utilization of evidence-based nursing practice [12, 13]. Appropriately implemented, the nursing process may provide meaning and relevance to professional knowledge [5]. Globally, the nursing process is recognized as an integral part of nursing education, practice, dynamic client care, and critical thinking in attempting to address the needs of clients [14]. The nursing process is the corner stone of the nursing profession [15, 16]. Using the nursing process as a tool to guide nursing care allows nurses to make independent and evidence-informed decisions that can encourage healing [17].

Utilization of the nursing process could assure nurses that they are meeting their responsibility for the patient care and enable evaluation of nursing care quality [18]. The essence of the nursing process lies in benefits to the client and nursing profession [19]. The nursing process guides nursing activities, promotes quality of care, and provides professional autonomy [20]. Substantial variations on the implementation of the nursing process across the globe have been reported. For instance, it has been reported 33.1% in Kenya [10], 57.1% in Nigeria [1], and 81.77% in Brazil [21]. On the other hand, a study conducted in the Democratic Republic of Congo showed that there was no implementation of the nursing process [22].

Factors that affect the implementation of the nursing process are complex and rooted in multiple factors. A review of several studies suggests that factors responsible to reduce the implementation of the nursing process include sociodemographic of nurses [10, 20, 22–29], patient-related factors [26, 28], knowledge and attitude of nurses [22, 26, 30, 31], and organizational factors [14, 23, 25, 29, 30]. On the other hand, a study conducted in Nigeria showed that institutional factors do not pose a barrier to the utilization of the nursing process [4, 32]. Identification of associated factors can be used as benchmarks to design appropriate measures, to improve client safety, and enhance utilization of resources.

Ethiopian Federal Ministry of Health has been engaged in improving quality of nursing care across the country in the last five years. Among these, national nursing process guideline was developed, national nursing mobilization activities were conducted, national dressing code guideline was launched, and national nursing service quality improvement audit tools were developed [33]. Nursing process is incorporated as the part of the curriculum to both in private and government education sector in Ethiopia. Though the government of Ethiopia gives emphasis on quality of healthcare service and nursing care. Nurses are paid 1500–2400 USD per year, and there is no retention strategies in most Ethiopian health institutions with high nurse turn over being a common challenge in the country. The nurse to patient ratio ranges from 1 : 6 to 1 : 12 based on the individual institution patient load and nurse availability.

Despite the effort of Ethiopian Federal Ministry of Health since 2011 to prepare and distribute protocol to the implementation of nursing process for all health care settings [34], the implementation of the nursing process in different health care setting is not well developed and organized [4, 26, 35]. In Ethiopia, nurses constitute the backbone of healthcare delivery system to improve the quality of health care service, and implementation of the nursing process may contribute a significant role. Different primary studies in Ethiopia [24–27] show the implementation of the nursing process as significant and a major issue in nursing care. However, variation was observed among these studies. Therefore, this study aimed to estimate the overall implementation of the nursing process and its association with the working environment and nursing knowledge in Ethiopia. Findings from the current study could serve as benchmark for institutional health care policymakers to implement appropriate measures to improve the implementation of the nursing process.

### 1.1. Research Questions

Three research questions were posited for this study:

1. What is the prevalence of implementation of the nursing process in Ethiopia?
2. What is the association between implementation of the nursing process and knowledge on nursing process?
3. What is the association between implementation of the nursing process and nurses' working environments?

## 2. Methods

### 2.1. Design and Search Strategy

To extract all relevant literature, electronic databases such as PubMed, Cochrane Library, Google Scholar, CINAHL, Cochrane Library, and Scopus were searched. In addition, a manual search of grey literature available on local university shelves, institutional repositories, and reference lists of all retrieved articles was conducted to identify additional relevant research to augment our meta-analysis. This search involved articles published from inception to April 1, 2019. The searches were restricted to full texts, free articles, human studies, and English language publications. Endnote X 8.1 reference manager software was used to collect and organize search outcomes and for removal of duplicate articles. The search strategy was developed using the Population Exposure Controls Outcome and Study design (PECOS) searching guide. The search was conducted using the following MeSH and free-text terms: “nursing process”, “implementation”, “nursing process implementation”, and “Ethiopia”. Boolean operators such as “AND” and “OR” were used to combine search terms.

### 2.2. PECOS Guide

#### 2.2.1. Population

All nurses working within health care settings for at least six months.

#### 2.2.2. Exposure

Nurses who have good knowledge on the nursing process and working within well-organized environments.

#### 2.2.3. Controls

Nurses who have poor knowledge on the nursing process and working in stressful environments.

#### 2.2.4. Outcome

Implementation of the nursing process.

#### 2.2.5. Study Design

All observational studies.

### 2.3. Eligibility Criteria

Studies were included if they met the following criteria: (1) articles conducted in Ethiopia; (2) articles published in peer reviewed journals and grey literature; (3) published in English language from inception to 2019; and (4) observational studies, reporting their outcome variable as implementation of the nursing process. Studies were excluded on any one of the following conditions: (1) not fully accessible (i.e., full text) at the time of our search process; (2) poor quality score as per the stated criteria; (3) duplicated citation; and (4) failure to measure the desired outcome (implementation of the nursing process).

### 2.4. Outcome of Interest

The main outcome of interest was the overall implementation of the nursing process. In the present review, implementation of the nursing process was evaluated either through nurse documentation of all its components from patient files or from the self-report of nurses working in a hospital or outpatient unit, in all of the following phases: data collection, nursing diagnosis, prescription of nursing, and evaluation of nursing [24, 27, 35]. The associated variables included in this review were working environment (i.e., well-organized versus stressful) and knowledge on the nursing process (i.e., good knowledge versus poor knowledge).

### 2.5. Data Extraction and Quality Assessment

Data were extracted by two authors using a Microsoft™ Excel spread sheet. For each included article, we extracted data regarding the name(s) of the author(s), year of publication, study area/region, health institution, study design, sample size, sampling technique, tool to measure the outcome, reported prevalence with its 95% confidence interval (CI), and information regarding the associated factors. The quality of each included study was assessed using the Newcastle–Ottawa Scale (NOS) [36]. Studies were included in the analysis if they scored ≥5 out of 10 points in three domains of modified NOS components for cross-sectional studies [36, 37]. The point allocation of each domain included selection (5 points), comparability (2 points), and outcome assessment (3 points). Furthermore, quality assurance checks were independently performed by three authors. Any disagreements at the time of data abstraction were resolved by discussion and consensus (Supplementary File 1).

### 2.6. Assessment of Risk of Bias in Included Studies

The risk of bias tool for prevalence studies developed by Hoy and colleagues [38] was used to assess the risk of bias among included studies. The risk of bias within the selected articles was classified as either low, moderate, or high. On the other hand, the Quality in Prognosis Studies tool was used to assess the risk of bias for studies, which reported the factors associated with the implementation of nursing process [39]. Both authors carried out the risk of bias assessment of the included studies independently (Supplementary File 2).

### 2.7. Heterogeneity and Publication Bias

Cochran's Q chi-square statistics and the *I*^2^ statistical test were conducted to assess the random variations between primary studies [40]. In this study, heterogeneity was interpreted as an *I*^2^ value of 0% = no heterogeneity, 25% = low, 50% = moderate, and 75% = high [41]. In case of high heterogeneity, subgroup analysis, meta regression, and sensitivity analyses were run to identify possible moderators of this heterogeneity. Potential publication bias was assessed by visually inspecting funnel plots and objectively using the Egger bias test (*p* < 0.05 was considered as statistical significant publication bias) [42].

### 2.8. Statistical Analysis

To obtain the overall implementation of the nursing process, a meta-analysis using the random effects DerSimonian and Laird model was performed due to significant heterogeneity among studies (*I*^2^ = 74.1%, *p* < 0.001) [43]. The pooled effect size (i.e., proportion and odds ratio (OR)) with a 95% confidence interval (CI)) was generated and presented using a forest plot. The meta-analysis was performed using the STATA™ Version 14 software [44]. Finally, for all analyses, *p* < 0.05 was considered statistically significant.

### 2.9. Presentation and Reporting of Results

To estimate the overall implementation of the nursing process, the preferred reporting items for systematic reviews and meta-analyses (PRISMA) guideline was used [45]. The PRISMA checklist was used alongside the final review. The entire process of study screening, selection, and inclusion were depicted with the aid of a flow diagram. Quantitative data were presented through forest plots and summary tables.

## 3. Results

### 3.1. Search Results

**Figure 1:**
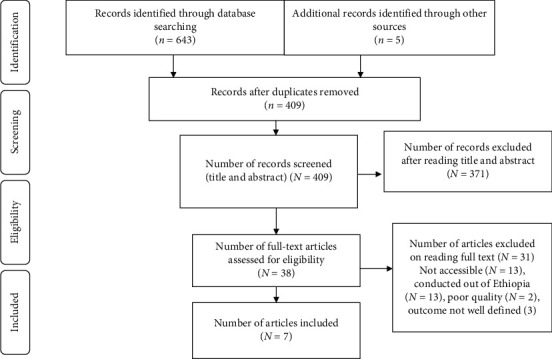
PRISMA flowchart diagram of the study selection.

The search strategy identified a total of 648 articles. About 643 studies were found from six international databases and the remaining 5 were through a manual search. The databases included PubMed (4), Scopus (83), PsycINFO (46), Cochrane Library (68), Google scholar (327), and CINAHL (115). Out of them, 239 duplicate records were identified and removed. Second, from the rest 409 impending article, 371 articles were excluded after reading of titles and abstracts based on the predefined eligibility criteria. Finally, 25 full text articles were read and assessed. Based on the predefined criteria and quality assessment, seven articles met eligibility for the review and were included in the final analysis (Figure 1).

### 3.2. Baseline Characteristic of the Included Studies

A total of seven studies with 1,268 study participants were included in this meta-analysis. The implementation of the nursing process was obtained from various regions across the country with two studies from Amharic region [26, 35], one each from Afar [24], Addis Ababa [25], Harare [28], Tigray [30], and Southern Nations, Nationalities, and People's Region (SNNPR) [27]. With respect to sample size, half the studies had fewer than 200 participants [24, 26, 27]. The highest implementation of the nursing process (52.1%) was reported in a study conducted in Addis Ababa [25], whereas the lowest (32.7%) was reported in a study conducted in SNNPR [27]. Regarding tools used to measure implementation of the nursing process, five studies [24–28] used self-report, and two studies [30, 35] employed a document review method. All the included studies were cross-sectional by design and were conducted among nurses working in different clinical setting of Ethiopia. The quality score of each primary study, based on the Newcastle–Ottawa quality score assessment, was moderate to high for all seven articles assessed (Table 1).

### 3.3. Implementation of Nursing Process in Ethiopia

**Figure 2:**
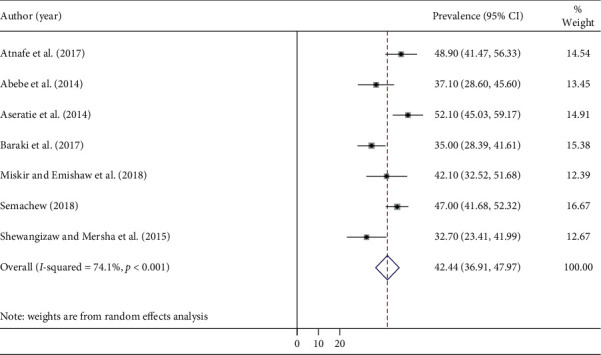
Forest plot showing the pooled prevalence of implementation nursing process.

The result of this meta-analysis using the random effects model showed that the overall implementation of the nursing process in Ethiopia was 42.44% (95% CI: 36.90, 47.97), with high significance of heterogeneity being observed (*I*^2^ = 74.1%; *p* < 0.001) (Figure 2).

### 3.4. Subgroup Analysis

**Figure 3:**
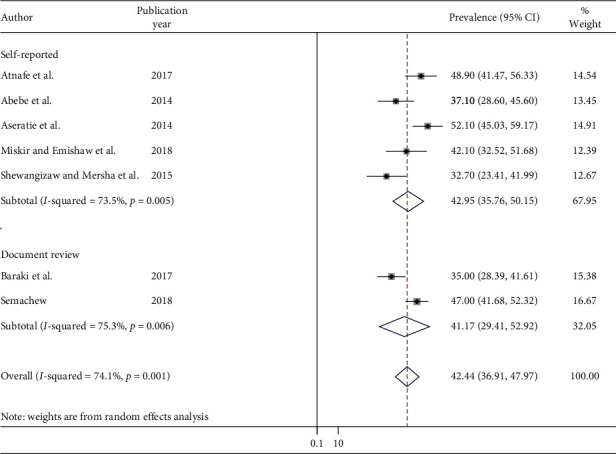
Subgroup analysis by methods of outcome measurement.

The presence of high significance heterogeneity among the primary studies requires the need to conduct subgroup analysis. As a result, to ascertain the sources of heterogeneity, we undertook a subgroup analysis using a type of outcome measure as the variable of interest. The finding of subgroup analysis using a type of outcome measure showed that the highest implementation of the nursing process was observed in studies conducted using self-reported methods 42.95% (95% CI: 35.76, 50.15) (Figure 3).

### 3.5. Meta-Regression Analysis

To investigate the possible source of variation across the included studies, we performed meta-regression by using publication year, outcome measurement, and sample size as covariate of interest. However, the result of the meta-regression analysis showed that both covariates were not statistically significant for the presence of heterogeneity (Table 2).

### 3.6. Sensitivity Analysis

**Figure 4:**
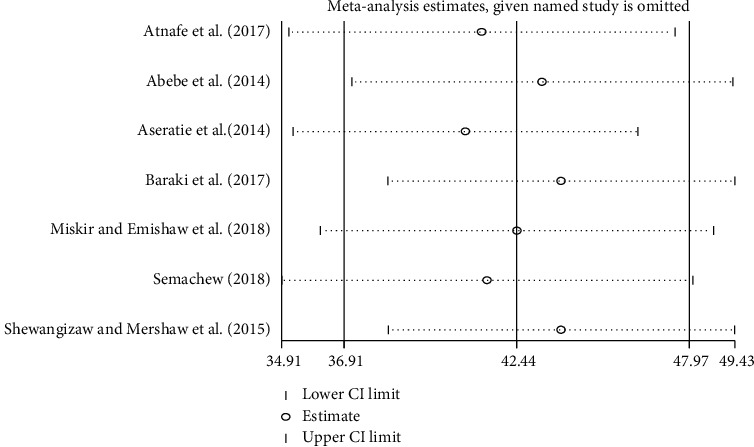
Result of sensitivity analysis of the seven studies.

To evaluate the effect of an individual study on the pooled effect size, sensitivity analysis was conducted. Sensitivity analyses using the random effects model revealed that no single study influenced the overall implementation of nursing process (Figure 4).

### 3.7. Publication Bias

To identify the presence of publication bias, Egger's test was performed. The evidence from Egger's regression test showed no significant proof of publication bias (*p*=0.349).

### 3.8. Association between Working Environment and Implementation of the Nursing Process

**Figure 5:**
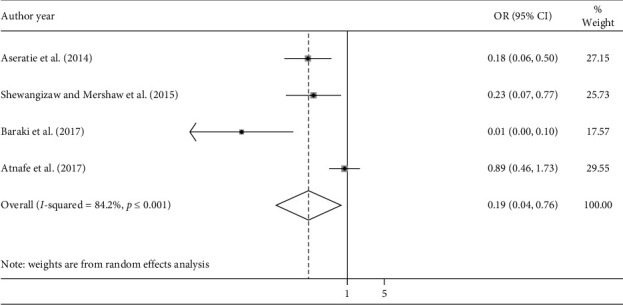
Forest plot showing the association between implementation of the nursing process and nurse working environment.

According to the current meta-analysis, those nurses working in a stressful environment were 81% less likely to implement the nursing process compared with nurses working in a well-organized environment (AOR = 0.19; 95% CI: 0.04, 0.76, *I*^2^ = 84.2%) (Figure 5). The evidence from Egger's regression test showed significant evidence of publication bias (*p*=0.032).

### 3.9. Association between Knowledge and Implementation of the Nursing Process

**Figure 6:**
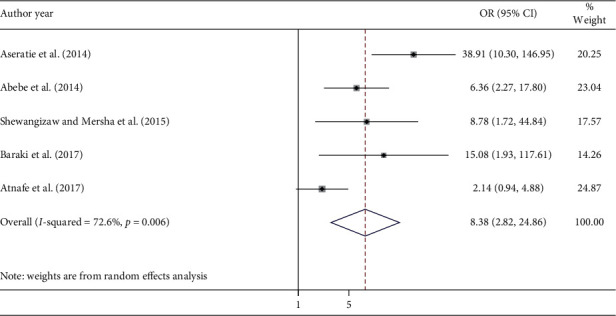
Forest plot shows the association between implementation of the nursing process and knowledge on nursing process.

Nurses with good knowledge were 8.38 times more likely to implement the nursing process compared with nurses having poor knowledge (AOR = 8.38; 95% CI: 2.82, 24.86) (Figure 6). The evidence from Egger's regression test showed that there was no publication bias (*p*=0.182).

## 4. Discussion

The main objective of this systematic review and meta-analysis was to estimate the overall implementation of the nursing process and its association with working environment and knowledge in Ethiopia. In this meta-analysis, the national pooled implementation of the nursing process in Ethiopia was estimated to be 42.44% (95% CI: 36.9, 47.9%). This finding was higher than that in a study conducted in Kenya with 33.1% [10]. However, this result was substantially lower than studies conducted in Nigeria with 57.1% [1] and Brazil with 81.77% [21]. This variation could be justified by difference in awareness, knowledge, educational background among nurses, policy, and health system strategies. For instance, in Brazil, there is an initiative, which emphasizes awareness-raising and training of nursing professionals in hospitals and outpatient clinics related to the nursing process implementation [31].

The result of the subgroup analysis based on methods of outcome measurement showed that the highest overall implementation of the nursing process was observed in studies using self-report technique 42.95% (95% CI: 35.76, 50.15). The present study revealed that nurses who had good knowledge of the nursing process were positively associated with implementation of the nursing process. This finding was supported by other studies conducted in developing and developed countries [46–49]. The possible explanation might be nurses who have theoretical knowledge on the nursing process could successfully promote quality of care to clients [20, 50].

According to the present review, nurses working in stressful environments were nearly 81% less likely to implement the nursing process as compared with those who are working in a well-organized environment. This finding is in agreement with a study conducted in Egypt [29]. This may reflect that a conducive environment is a necessary condition for effective and efficient nursing practice.

The meta-analysis conducted in this study has limitations that should be considered in future research. First, it is difficult to determine if the results from various regions are representative of the entire country, as no data were found for all regions of Ethiopia; second, most of the studies included had small sample size. Third, it was challenging to synthesise some of the factors as they were not defined or measured in the same way across the different studies; fourth, included studies only reported on hospital level data. Last, it was challenging to compare and contrast our findings with others because of lack of other published systematic review and meta-analysis on the implementation of the nursing process.

### 4.1. Implications for Nursing Practice

This meta-analysis has implications for clinical practice. Estimating the overall implementation of the nursing process would serve as a baseline for health care providers on the utilization of the nursing process, as standard of care, and to address client demand. The finding emphasizes the need for nursing educators to facilitate and encourage knowledge of the nursing process amongst their students in order to embed this practice. Furthermore, there is an imperative to design and implement different strategies on nursing knowledge and working environment to enhance the potential implementation of the nursing process across the health care system.

## 5. Conclusion and Recommendations

The overall implementation of the nursing process in Ethiopia was relatively low. Good knowledge of the nurse had paramount benefits to improve implementation of the nursing process. Therefore, nurses can be educated on the imperative of knowledge in order to enhance the nursing process implementation and to improve the overall quality of healthcare services. Furthermore, policymakers (FMOH) and other concerned bodies should give special attention to improve implementation of the nursing process.

## Figures and Tables

**Table 1:** Baseline characteristics of studies included in the meta-analysis.

Primary author	Pub. year	Study area, Region	Health facility name	Sampling	Sample size	Prevalence % (95% CI)	Fool to measure outcome variable	Quality score
Abebe et al. [26]	2014	Amhara	Finoteselam and Debre Markos Hospital	Census	139	37.1 (28.6–45.6)	Self-reported	6
Miskir and Emishaw et al. [24]	2018	Afar	Afar region hospitals	Simple random	107	42.1 (32.5–51.6)	Self-reported	7
Aseratie et al. [25]	2014	Addis baba	Public hospitals	Simple random	202	52.1(45.0–59.2)	Self-reported	8
Shewangizaw and Mersha et al. [27]	2015	Arba Minch, SNNPR	Arba Minch General Hospital	Simple random	105	32.7 (23.4–41.9)	Self-reported	8
Baraki et al. [30]	2017	Tigray	Hospitals of Central and Northwest zones	Simple random	200	35.0 (28.4–41.6)	Document review	7
Semachew [35]	2018	Amhara	Felege Hiwot Referral Hospital Debretabor and Finoteselam general hospitals	Systematic random	338	47.0 (41.7–52.3)	Document review	7
Atnafe et al. [28]	2017	Harare	Public Hospitals of Harari People National Regional State	Systematic random	177	48.9 (41.5–56.3)	Self-reported	6

**Table 2:** Meta regression analysis for the included studies to identify source of heterogeneity.

Covariate (source)	Coefficients	Standard error	*p* value	95% CI
Publication year	−0.010	1.011	0.992	−6.062, 5.131
Sample size	0.047	0.043	0.338	−0.073, 0.168
Type of outcome measure Self-report Document review (ref.)	0.029	1.002	0.978	−2.546, 2.605

## Data Availability

The data used to support the findings of this study are included within the article.

## Abbreviations

CI: Confidence interval
FMOH: Federal ministry of health
OR: Odds ratio
PRISMA: Preferred reporting items for systematic reviews and meta-analyses
SNNP: Southern nations, nationalities, and peoples
WHO: World Health Organization.

## Supplementary Materials

Supplementary Materials Supplementary file 1: methodological quality assessment of cross-sectional studies using the modified Newcastle–Ottawa Scale (NOS). Supplementary file 2: risk of bias tool for implementation of the nursing process and its association with working environment and knowledge in Ethiopia.
