# Supplementary material for: Implementation of Nursing Process and Its Association with Working Environment and Knowledge in Ethiopia: A Systematic Review and Meta-Analysis
Source: Nurs Res Pract. 2020 Jul 18;2020:6504893. doi: 10.1155/2020/6504893 (PMC7383312; doi:10.1155/2020/6504893)
Supplement: Supplementary Materials — Supplementary file 1: methodological quality assessment of cross-sectional studies using the modified Newcastle–Ottawa Scale (NOS). Supplementary file 2: risk of bias tool for implementation of the nursing process and its association with working environment and knowledge in Ethiopia. [file 6504893.f1.docx]

Supplementary file 1 : Methodological quality assessment of cross-sectional studies using modified Newcastle - Ottawa Scale (NOS)

| **First author, publication year** | Criteria | | | | | | | |  |
| --- | --- | --- | --- | --- | --- | --- | --- | --- | --- |
|  | **Selection** | | | | **Comparability** | | **Outcome** | |  |
|  | **Representativeness of the sample** | **Sample size** | **Non –respondents** | **Ascertainment of exposure/risk factor** | **The study controls for the most important factor** | **The study control for any additional factor** | **Assessment of the outcome** | **Statistical test** | **Total score**  **(10)** |
| Abebe N etal 2014 | **B*** | **B*** | **A*** | **A*** | **B*** | **A*** | A* | **A*** | **6** |
| Miskir Y etal 2018 | **A*** | **A*** | **A*** | **A*** | **A*** | **B*** | A* | **A*** | **7** |
| Aseratie M etal 2014 | **A*** | **A*** | **A*** | **A*** | **A*** | **B*** | A* | **A*** | **8** |
| Shewangizaw Z etal 2015 | **A*** | **A*** | **A*** | **B*** | **A*** | **A*** | A* | **A*** | **8** |
| Baraki Z etal,2017 | **B*** | **A*** | **A*** | **B*** | **A*** | **A*** | A* | **A*** | **7** |
| Semachew Ayele 2018 | **A*** | **A*** | **A*** | **A*** | **-** | **B*** | A* | **A*** | **7** |
| Atnafe G etal ,2017 | B* | **B*** | **A*** | **A*** | **A*** | **-** | A* | **A*** | **6** |

*Note: from each item account point. (Accept the study if total score ≥5)*

Selection: (Maximum 5 stars)
1) Representativeness of the sample: a) Truly representative of the average in the target population. * (all subjects or random sampling) .b) Somewhat representative of the average in the target population. * (nonrandom sampling) .c) Selected group of users.d) No description of the sampling strategy.
2) Sample size:a) Justified and satisfactory. *.b) Not justified.
3) Non-respondents: a) Comparability between respondents and non-respondents characteristics is
established, and the response rate is satisfactory. * .b). The response rate is unsatisfactory, or the comparability between respondents
and non-respondents is unsatisfactory. c) No description of the response rate or the characteristics of the responders and
the non-responders.
4) Ascertainment of the exposure (risk factor): a) validated measurement tool. ** .b) Non-validated measurement tool, but the tool is available or described.* c) No description of the measurement tool.
Comparability: (Maximum 2 stars)
1) The subjects in different outcome groups are comparable, based on the study design or analysis. Confounding factors are controlled. a) The study controls for the most important factor (select one). * b) The study control for any additional factor. *
Outcome: (Maximum 3 stars)
1) Assessment of the outcome: a) Independent blind assessment. **,b) Record linkage. **,c) Self report. *,d) No description.

2) Statistical test:a) The statistical test used to analyze the data is clearly described and appropriate, and the measurement of the association is presented, including confidence intervals and the probability level (p value). *,b) The statistical test is not appropriate, not described or incomplete

Supplementary file 2: Risk of Bias Tool for implementation of the nursing process and its association with working environment and knowledge in Ethiopia.

| Name of author/year of pub. | Target population a close representation of the national pon. | Sampling frame a true or close representation of the target pon. | random selection used to select the sample | non-response bias minimal | data collected directly from the subjects | acceptable case definition used in the study | reliability and validity of instrument | Was the same mode of data collection used for all subject | Was the length of the shortest prevalence period for the parameter of interest appropriate. | the numerator( s) and denominato r(s) for the parameter of interest appropriate | Summary item on the overall risk of study bias |
| --- | --- | --- | --- | --- | --- | --- | --- | --- | --- | --- | --- |
| Abebe N etal,2014 | low risk | low risk | high risk | low risk | low risk | low risk | low risk | low risk | high risk | low risk | Low risk |
| Miskir Y etal,2018 | low risk | low risk | low risk | low risk | low risk | low risk | low risk | low risk | high risk | low risk | Low risk |
| Aseratie M etal,2014 | low risk | low risk | low risk | low risk | low risk | low risk | low risk | low risk | Low risk | low risk | Low risk |
| Shewangizaw Z etal,2015 | low risk | low risk | high risk | low risk | low risk | low risk | low risk | low risk | low risk | low risk | Low risk |
| Baraki Z etal,2017 | low risk | low risk | high risk | low risk | low risk | low risk | low risk | low risk | low risk | low risk | Low risk |
| Semachew Ayele,2018 | low risk | low risk | high risk | low risk | low risk | low risk | low risk | low risk | low risk | low risk | Low risk |
| Atnafe G etal,2017 | low risk | low risk | High risk | low risk | low risk | low risk | low risk | low risk | high risk | low risk | Low risk |

- Note :
- LOW RISK OF BIAS: Further research is very unlikely to change our confidence in the estimate.
- MODERATE RISK OF BIAS: Further research is likely to have an important impact on our confidence in the estimate and may change the estimate.
- HIGH RISK OF BIAS: Further research is very likely to have an important impact on our confidence in the estimate and is likely to change the estimate.
